# Use of hypnosis to treat chronic somniloquy (sleep talking): a Case Report

**DOI:** 10.3389/frsle.2026.1707583

**Published:** 2026-04-13

**Authors:** Melvin S. Marsh

**Affiliations:** 1 After Hours Hypnotherapy, Augusta, GA, United States; 2 Department of Psychology, Alabama State University, Montgomery, AL, United States

**Keywords:** behavioral intervention, case study, clinical hypnosis, hypnotherapy, sleep disorders, sleep talking, somniloquy, treatment outcomes

## Abstract

**Introduction:**

This case study describes a hypnotherapeutic intervention for a 46-year-old man with chronic somniloquy that had persisted for decades, worsening during periods of stress. The patient sought treatment due to spousal complaints regarding the disturbance in her sleep.

**Methods:**

A treatment plan consisting of eight bi-weekly hypnotherapy sessions was developed and initiated. The intervention focused on clinical hypnosis as the primary therapeutic modality for addressing somniloquy.

**Results:**

The intervention yielded positive results. By the sixth scheduled session, the patient's wife reported that his sleep talking had been nearly completely eliminated, leading to an early discontinuation of treatment.

**Discussion:**

This case study suggests that clinical hypnosis may be a viable treatment option for chronic somniloquy. Further research is warranted to explore this therapeutic approach on a larger scale.

## 1 Introduction

Somniloquy, or sleep talking, is a common occurrence in children, with current estimates suggest 50% of children speak in their sleep at some point in their childhoods, and only approximately 5% of adults still maintain this behavior (Jha and Jha, 2024; Alfonsi et al., 2019). The quality and quantity of the speaking can vary and can range from a few words to a full, but one-sided conversation (Jha and Jha, 2024). While generally harmless, it can lead to a decreased quality of sleep for those affected by such a disorder, both direct sufferers and their bed partners, who may be woken up by incessant high-quality talking.

More known as entertainment than as a treatment, hypnosis, also known as hypnotherapy, as a psychological and medical treatment, goes back to Ancient Egypt (Reid, 2016). But what is hypnosis? American Psychological Association's Division 30 Society of Psychological Hypnosis, which defines hypnosis as “a state of consciousness involving focused attention and reduced peripheral awareness characterized by an enhanced capacity for response to suggestion” (Elkins et al., 2015).

Dr. Milton H. Erickson (1901–1980), an American psychiatrist, is perhaps the most famous practitioner of indirect hypnosis (Saudi, 2005). Ericksonian style hypnosis is typically unscripted and individualized to the client, often utilizing the client's past knowledge and experience, additionally asking the question “What is missing for the client?” and assuming the subconscious is able to devise possible solutions to the situation at hand (McNeilly, 2000). The use of Ericksonian hypnosis requires greater fluidity and nuance from the clinician. The author/therapist has extensive training and experience in this technique.

While there are reviews on the use of hypnosis in the treatment of non-sleep talking parasomnias (Solelhac et al., 2026; Hauri et al., 2007), here is little information in the current literature about treatments for sleep talking, much less the role any form of clinical hypnosis might play in reducing this. This study appears to be the first to address sleep talking in the hypnosis literature, although there might be cases in the Ericksonian archives, as Erickson had extensive experience treating other sleep disorders, such as insomnia (McNeilly, 2000).

## 2 Patient background

Patient, Mr. N, was a married 46-year-old white man with chronic somniloquy from which he had suffered for decades. As this is a medical hypnotherapy practice that does not take self-referrals, he was referred to the practice by a licensed mental health provider for this issue as a “last resort.” Mr. N could not report the age at which it began, although it was believed to have started in youth. As he frequently did not remember talking in his sleep, he had to rely on family members. Family members reported increased sleep-talking episodes during times that Mr. N associated with higher stress levels. While he had been experiencing this for decades, the patient reported to therapy due to spousal complaints that he was waking her up several times a night, with no particular times being more or less likely. His medical and psychological history was unremarkable, and he did not have any diagnosed conditions, sleep-related or otherwise. Other treatments for this condition, including cognitive behavioral therapy as well as medication, had been attempted by other providers. Patient denied excessive drug or alcohol use or any pertinent medical history that could influence any form of sleep disorder. He believes he may have attention deficit disorder, but it is unclear if this was professionally diagnosed, as otherwise, he had no other diagnoses. He denied taking any medications. He reported two primary sources of stress, which include a family member with a chronic illness and job changes that came about due to the COVID-19 pandemic.

## 3 Methods: Ericksonian hypnotherapy

After consultation in November 2020, a treatment plan consisting of eight 1-h-long hypnotherapy sessions scheduled every 2 weeks was devised. As the author has over 1,500 h of hypnosis training and has an area of interest in Ericksonian hypnosis, this individualized form of hypnosis was utilized. Furthermore, the author also has certifications and training specifically in Ericksonian hypnosis for treating sleep disorders, although this did not include talking specifically.

### 3.1 Sessions

#### 3.1.1 Session 1—December 2020

This session served as an introduction to hypnosis and hypnotherapy, as he had not reported previous experience with hypnosis of any form. After a progressive relaxation induction and a 5-to-0 deepener/fractionation combination, he was asked to visualize a peaceful place and another version of himself to ask why he constantly spoke in his sleep, and that it was ok to be quiet from time to time. Upon reorienting, Mr. N said he had some ideas of reasons why what needed to be done based on what the “other” Mr. N reported.

#### 3.1.2 Session 2—January 2021

Instead of 2 weeks after his first session, this took place a month after due to scheduling issues. Regardless, Mr. N said his sleep talking had reduced per his wife, but that her request was for something she could say to him to get him to stop talking and go back to sleep. His induction was based on remembering his previous trance experience and using the same deepener. His trance primarily focused on the idea that it was ok to be quiet, and his visualization primarily focused on being in a library with a librarian going “Shhhh,” and that if he heard “Shhhh,” he would go back to sleep.

#### 3.1.3 Session 3—February 2021

Mr. N reported doing “really good” and relayed that his wife said his sleep-talking had “decreased dramatically.” Interestingly, he reported that his wife claimed he was much nicer when she told him he was talking in his sleep and would apologize instead of being combative, as he had been prior. He reported he was still experiencing stress at work, and his friends reported that he was too hard on himself. Session focused on using the relaxation he experienced in trance to be his “new normal” and treating himself as well as he treats others.

#### 3.1.4 Session 4—February 2021

Mr. N emailed a few days prior to the appointment, stating that his relationship with work has improved, and his talking has continued to decrease. However, his wife reported that most of his sleep talking involved him waking her to ask if she was ok. Trance involved focusing on self-care and not bothering his wife until she asks for help.

#### 3.1.5 Session 5—March 2021

Mr. N returned to the session, stating that although he does not seem to be talking distinctly anymore, he is still making a lot of grunting noises, which he claims he makes when thinking. He wondered if it might be “some type of praying situation.” His trancework involved praying across cultures and how it is perfectly acceptable to pray whenever he likes before bed, so that he does not need to pray when he is asleep.

#### 3.1.6 Session 6—March 2021

While two additional appointments were scheduled, Mr. N came to the appointment claiming that his wife said he only woke her up once during the previous 2 weeks, and so he believed he could cancel the next two appointments. No hypnosis or interventions were offered during this session, so this served more as a debriefing. He stated he loved his job now, noting that very little had changed since treatment began, and he felt inspired to renew a prior professional license he held. He again confirmed he was willing to allow his case to be written up, as it appears to have been successful.

### 3.2 Patient perspective

Mr. N reported being very impressed by how much happier and more content he was, as well as by his wife's report that he spoke much less throughout the night. He reported wanting to learn hypnosis so he could provide similar services if he returned to being a licensed provider.

## 4 Discussion

While there is very little information on the treatment of sleep talking in the literature, in general, much less with hypnosis, hypnosis has been used to alleviate other parasomnias, including parasomnia overlap disorder (Kohler et al., 2015), insomnia (Lam et al., 2018) as well as other parasomnias, including sleepwalking and nightmares (Hauri et al., 2007). It should be noted that in Kohler et al. (2015), there was a small amount of sleep talking, which did get resolved; however, it was considered to be a part of the parasomnia overlap disorder and not purely sleep talking. Given Mr. N had been experiencing sleep talking for decades, and it started to decrease after one hypnotherapy session, it seems unlikely that there was a case of spontaneous recovery.

The success of this intervention likely stems from the Ericksonian principle of utilizing the patient's own subconscious resources to resolve the “missing” quietude in his sleep. By facilitating a dialogue with an “other” version of himself, hypnotherapy bypassed conscious resistance and empowered Mr. N to identify internal regulatory solutions that previous clinical attempts had failed to reach. The treatment effectively lowered his baseline hyper-arousal, shifting his “new normal” from a state of work-related stress and self-criticism to one of deep relaxation and self-care. Furthermore, the introduction of a specific post-hypnotic trigger—the spousal “Shhhh” cue provided a functional bridge between the hypnotic state and his domestic environment, retraining the brain to inhibit speech muscles upon hearing the auditory signal. Ultimately, the transition from combative sleep talking to apologetic responses, and eventually to silence, suggests that hypnotherapy did not merely suppress a symptom, but fundamentally restructured the affective tone of his parasomnia and allowed him to categorize cognitive processes, such as “praying,” before sleep began.

The drawbacks of this case include that the sample size was simply *n* = 1, and hypnotizability suggestibility scale data were not collected, as this is not a standard part of the practice's intake, though Mr. N appeared to go into trance quickly and effortlessly. More studies will need to be performed to further understand the nature of treating sleep talking with hypnosis.

## 5 Conclusion

The patient initially met the diagnostic criteria for chronic somniloquy and had been for some time. At the time of the consultation, the patient's sleep talking would wake up his wife several times a night, while he had little to no memory of waking. After the first session, the patient's wife stated that while he did speak, she could already notice a reduction. By the end of the fifth hypnosis session, the patient's wife stated that he had only woken her up once in the 2 weeks prior to session 6. This would suggest that hypnosis might be an appropriate treatment for excessive talking in one's sleep.

## Data Availability

The datasets presented in this article are not readily available because of ethical and privacy restrictions. Requests to access the datasets should be directed to the corresponding author.
